# Prevalence and antimicrobial susceptibility profile of *Salmonella* and *Shigella* among food handlers working in food establishment at Hawassa city, Southern Ethiopia

**DOI:** 10.1186/s13104-019-4725-y

**Published:** 2019-10-30

**Authors:** Neja Awol, Demiss Nigusse, Musa Ali

**Affiliations:** 1 Worabe Comprehensive Specialized Hospital, Silte Zone, Worabe, Ethiopia; 2 School of Medical Laboratory Science, College of Medicine and Health Science, Hawassa University, Hawassa, Ethiopia

**Keywords:** Food handlers, Prevalence, Antibiotic susceptibility, *Salmonella*, *Shigella*, Hawassa, Ethiopia

## Abstract

**Objective:**

The aim of this study was to determine the prevalence of *Salmonella* and *Shigella*, antibiotic susceptibility profile and associated factors among food handlers working in food establishment from June to December 2018 at Hawassa city, Southern Ethiopia.

**Results:**

Out of the 236 food handlers screened for stool culture, 5 (2.12%) were positive for *Salmonella* species and all of them were negative for *Shigella* species. All *Salmonella* species isolated were susceptible to ciprofloxacin and ceftriaxone but among the five isolated *Salmonella* species, 4 (80%), 3 (60%), 2 (40%), 2 (40%), and 2 (40%) were resistant to tetracycline, ampicillin, amoxicillin–clavulanic acid, cotrimoxazole, and chloramphenicol respectively. Only food handlers hand washing the habit after toilet had a significant association with the prevalence of *Salmonella* species (P = 0.03).

## Introduction

Food-borne disease (FBD) remains a major public health problem across the globe. Several reports have shown that poor personal hygiene and handling of food stuffs could lead to various illnesses. Center for Disease Control and Prevention (CDC) identified more than 400 food-related infections, among which 20% are due to food handlers [1]. The spread of disease through food handlers is a common and persistent problem worldwide and food handlers may be carrying a wide range of enteric pathogen and have implicated in the transmission of many infections to the public [2].

The FBD is defined as diseases that are generally either infectious or toxic in nature and caused by the agents that enter the body through the ingestion of food [3]. The main causes of FBDs are bacteria (66%), chemicals (26%), virus (4%) and parasites (4%) [4, 5].

Food-borne diseases are a serious threat to people in Africa, causing an unbearable public health burden and massive economic losses [6]. From the World Health Organization (WHO) reports, more than 91 million people in Africa region fall ill each year from FBD; resulting in 137,000 deaths. Diarrheal diseases are responsible for 70% of the burden of FBDs in the region, particularly non-typhoidal *Salmonella*, *E. coli*, and Food-borne cholera [7]. In developing countries, 70% of cases of diarrheal disease are associated with the consumption of contaminated food [8].

A food handler is anyone who works in a food and drink establishments and who handles food/have contact with any equipment or utensils that are likely to be in contact with food, such as plates, or chopping boards [9]. Food handlers who harbor and excrete entero-pathogenic bacteria may contaminate foods from their feces via their fingers, then to the food processing, and finally to healthy individuals [10].

There are more than 250 different FBDs in the world and most are caused by a variety of bacteria, viruses, and parasites. Food and water can be contaminated by bacteria such as *Salmonella*, *Campylobacter*, *Listeria*, *pathogenic Escherichia coli*, *Yersinia*, *Shigella*, *Enterobacter*, and *Citrobacter* [11, 12].

National Hygiene and Sanitation Strategy program reported that about 60% of the disease burden is related to poor hygiene and sanitation in Ethiopia. According to patient morbidity statistics (Hospitals and Health centers) of selected food-borne and food-related cases, the annual incidence of food-borne illnesses in Ethiopia ranged from 3.4 to 9.3%, the median being 5.8% for the years 1985/86 to 1989/90 [13, 14].

The aim of the current study was to determine the prevalence and antimicrobial susceptibility profile of *Salmonella* and *Shigella* species among food handlers working in food establishments at Hawassa city, Southern Ethiopia.

## Main text

### Methods

#### Study area

Hawassa is the capital city of Southern Nations Nationalities and Peoples Region (SNNPR) which is located on the shores of Lake Hawassa in the Great Rift Valley and located 275 to south of Addis Ababa, a capital city of Ethiopia. The Ethiopian Central Statistical Agency (CSA 2007), gives the estimated population of Hawassa for 2007 as 259,803.

#### Study design and study period

A community-based cross-sectional study was conducted from June to December 2018 among food handlers working in food establishments at Hawassa city, Southern Ethiopia.

#### Sample size determination

The sample size was calculated by using a single population proportion formula.

$${\text{n}} = {\text{Z}}^{ 2} \times {\text{P}} \times \left( { 1- {\text{P}}} \right)/{\text{D}}^{ 2}$$

where n is the sample size; Z is the reliability coefficient (confidence level) which is 95% = 1.96, P is the anticipated population proportion, D is the margin of error, which is 4% = 0.04.

By using the anticipated population proportion of 10% from a study in Arba Minch, South Ethiopia [15],

$${\text{n}} = 1. 9 6^{ 2} \times \left( {0. 1} \right) \times \left( { 1- 0. 1} \right)/0.0 4^{ 2} = 2 1 7,$$

after adding 10% of non-respondents the final sample size was = 217 + 22 = 239.

#### Sampling technique

Simple random sampling technique was used to select the 120 food establishments among the 358 licensed food establishments found in the Hawassa city. From each food establishments, two food handlers were selected by simple random sampling technique.

#### Data collection procedure

Data related to socio-demographic characteristics, hand washing habit, and food safety practices of food handlers were collected by face-to-face interview using pre-tested structured questionnaire and by observation.

#### Sample collection, transport and processing

Stool samples were collected in labeled, clean, dry and leak**-**proof cups. The specimens were immediately placed on to Cary-Blair transport medium using swabs and transported in ice- packed box to SNNPR, Hawassa public health regional laboratory.

About 1 g of stool sample was directly inoculated on to MacConkey (MAC) and xylose-lysine-deoxycholate (XLD) agar plates by using a sterile wire loop. The inoculated culture media were incubated at 37 °C for 18–24 h and examined for colony characteristics of *Salmonella*/*Shigella* species. The growths of suspected colonies of *Salmonella*/*Shigella* species were detected by their characteristic appearance on MAC agar (non-lactose fermenter, smooth, colorless to yellow colonies, sometimes with black centered) and on XLD agar (small pin to red colonies and black-centered colonies [16].

For further confirmation, typical and suspected colonies of *Salmonella*/*Shigella* were selected and streaked onto the surface of nutrient agar. Colonies were picked from the streaked nutrient agar and inoculated into Triple Sugar Iron (TSI) agar, Simmon’s citrate agar, urea broth, hydrogen sulfide (H2S) production, indole production and motility in Sulfide–Indole–Motility (SIM) medium and incubated for 24 h at 37 °C [17]. Colonies producing an alkaline slant (red color) with acid butt (yellow color) on TSI with H2S, positive for lysine (purple color), positive for citrate utilization, positive for motility, negative for urea hydrolysis (red color) and negative for tryptophan utilization (indole test) (yellow–brown ring) were considered to be *Salmonella* species.

#### Antimicrobial susceptibility testing

Antimicrobial susceptibility test was determined by using disc diffusion method [17]. Briefly, pure identified colonies from the overnight culture were suspended in nutrient broth and incubated for 4 h at 37 °C. Turbidity of broth culture was checked against 0.5 McFarland standards. By using sterile cotton swab the organism in the broth was uniformly inoculated onto Mueller–Hinton Agar. The antimicrobial agents used for the isolates were amoxicillin-clavulanic acid (30 μg), ampicillin (10 μg), chloramphenicol (30 μg), ciprofloxacin (5 μg), ceftriaxone (30 μg), nalidixic acid (30 μg), tetracycline (30 μg) and cotrimoxazole (25 μg). The zones of inhibition were read and results were examined for the diameter of growth inhibition around the discs and interpreted as sensitive, intermediate or resistant according to CLSI, 2018 guideline [18].

#### Data management and quality control

The qualities of data were assured by proper designing and pre-testing of the questionnaires. The questionnaire was pre-tested on 5% of the sample at Hawassa University main campus) who are not included in the study. As quality control, *E. coli* (ATCC 25922) was used. Sterility of culture media was checked by incubating 5% of the batch at 35 °C overnight and was observed for bacterial growth.

#### Data processing and analysis

Data were entered, cleaned, coded and analyzed using statistical package for social science (SPSS) version 23. Data were organized, summarized, and presented in descriptive statistical methods. A *P*-value of ≤ 0.05 was considered as statistically significant association between the factors and *Salmonella/Shigella* species by using Chi-square/Fishers test.

### Results

#### Sociodemographic characteristics

A total of 236 food handlers serving in food establishments found in Hawassa city participated in the current study with a response rate of 98.75%. Of these, 150 (63.6%) were females and the mean age was 23.8 years ± 10.48. Majority 125 (53%) of the study participants were in the age group between 21 and 30 years. Of the total participants 157 (66.5%) were served as cookers.

Out of the total study participants 178 (75.4%), 84 (35.6%), 162 (68.6%), 88 (37.3%) washed their hands with water and soap before touching food, after touching dirty materials, and after touching body parts respectively. Eighty-eight (37.3%) of participants attended medical checkup (Table 1).

**Table 1:** Hand washing and food safety practices of food handlers working at Hawassa city, Southern Ethiopia, June to December 2018 (N = 236)

Variables	Category	Frequency	Percent
Wash hand before touching food	With water and soap	178	75.4
	Only with water	58	24.6
Wash hand after touching dirty materials	With water and soap	84	35.6
	Only with water	120	50.8
	Not wash at all	32	13.6
Wash hand after touching body parts	With soap and water	38	16.1
	Only with water	32	13.6
	Not at all	166	70.3
Wash hand after toilet	With soap and water	162	68.6
	Only with water	74	31.4
Use hand glove	Yes	13	5.5
	No	223	94.5
Attend medical checkup	Yes	88	37.3
	No	148	62.7
Wear hair cover	Yes	91	38.6
	No	145	61.4
Wear gown or coat	Yes	194	82.2
	No	42	17.8
Gown or coat clean	Yes	101	42.8
	No	135	57.2
Finger nails trimmed	Yes	147	62.3
	No	89	37.7
Painted nail	Yes	21	8.9
	No	215	91.1
Wear jewelry	Yes	54	22.9
	No	182	77.1

#### Prevalence of *Shigella* and *Salmonella*

Among 236 food handlers participated in the current study, 5 (2.1%) were positive for *Salmonella* species, while no *Shigella* species were isolated from the stool samples.

#### Factors associated with the prevalence of *Salmonella* species among food handlers

Among different variables assessed in this study, only washing hands after toilet was significantly associated with prevalence of *Salmonella* species (Table 2).

**Table 2:** Association of *Salmonella* species with socio-demographic factors, hand washing habit and food safety practices of food handlers at Hawassa city, Southern Ethiopia, June to December 2018 (N = 236)

Variables	Categories	*Salmonella* species(n = 5)			P value
		Positive n (%)	Negative n (%)	Total	
Gender	Male	1 (1.2)	85 (98.8)	86	0.322
	Female	4 (2.7)	146 (97.3)	150	
Educational status	Illiterate	–	20 (100)	20	0.492
	Literate	5 (2.3)	211 (97.7)	216	
Trained on food preparation	Yes	–	21 (100)	21	1.00
	No	5 (2.3)	210 (97.7)	215	
Washed hand before touching food	Soap and water	2 (1.1)	176 (98.9)	178	0.097
	Water only	3 (5.2)	55 (94.8)	58	
Washed hand after toilet	Soap and water	–	162 (100)	162	0.03
	Water only	5 (6.8)	69 (93.2)	74	
Medical checkup	Yes	1 (1.1)	87 (98.9)	88	0.653
	No	4 (2.7)	144 (97.3)	148	
Worn hair cover	Yes	1 (1.1)	91 (98.9)	92	0.651
	No	4 (2.8)	140 (97.2)	144	
Worn gown or coat	Yes	5 (2.6)	189 (97.4)	194	0.589
	No	–	42 (100)	42	
Trimmed finger nails	Yes	2 (1.4)	145 (98.6)	147	0.368
	No	3 (3.4)	86 (96.6)	89	
Worn jewelry	Yes	2 (3.7)	52 (96.3)	54	0.322
	No	3 (1.6)	179 (98.4)	182	
Use hand glove	Yes	1 (7.7)	12 (92.3)	13	0.249
	No	4 (1.8)	219 (98.2)	223	
Wash hand after touching body parts	Soap and water	–	38 (100)	38	0.593
	Water only	1 (3.1)	31 (96.9)	32	
	No	4 (2.4)	162 (97.6)	166	
Wash hand after touching dirty materials	Soap and water	–	84 (100)	84	0.085
	Water only	5 (4.2)	115 (95.8)	120	
	No	–	32 (100)	32	

#### Antimicrobial susceptibility test of *Salmonella* species

Out of five *Salmonella* species 4 (80%), 3 (60%), 2 (40%) were resistant to tetracycline, ampicillin, and cotrimoxazole respectively (Table 3).

**Table 3:** Antimicrobial sensitivity patterns of *Salmonella* species isolates from selected food handlers in Hawassa city, South Ethiopia June to December 2018

Antibiotics	Susceptible	Intermediate	Resistant
	n (%)	n (%)	n (%)
Ciprofloxacin	5 (100)	–	–
Tetracycline	1 (20)	–	4 (80)
Ampicillin	2 (40)	–	3 (60)
Amoxicillin/clavulanic acid	2 (40)	1 (20)	2 (40)
Ceftriaxone	5 (100)	–	–
Cotrimoxazole	3 (60)	–	2 (40)
Chloramphenicol	2 (40)	1 (20)	2 (40)
Nalidixic acid	4 (80)	–	1 (20)

### Discussion

In the present study, from the total 236 food handlers 5 (2.1%) of them were positive for *Salmonella* species which is in line with the findings from Bahir Dar, Northern Ethiopia (2.7%) [19], Ghana (2.3%) [20] and Gondar, Ethiopia (1.3%) [21]. However, the prevalence of *Salmonella* species in the present study is lower than the prevalence reported from Nigeria (5.5–17%) [22, 23], Arba Minch, Southern Ethiopia (6.9%) [15], Saudi Arabia (3.86%) [24] and Haramaya, Eastern Ethiopia (3.6%) [25]. The finding of current study is high compared to report from Hawassa, Ethiopia [26], Gondar, Ethiopia [27], Makah, Saudi Arabia [28], Jordan [29], Thailand [30], Japan [31], Dilla, Southern Ethiopia [32] and Nigeria [2]. The difference in the prevalence of *Salmonella* species among food handlers observed across different countries and within in country may be due to laboratory methods used, level of education, economic status, and characteristics of study participants.

In the present study no *Shigella* species were recovered from the stool culture which is similar with the results from Addis Ababa [33], Saudi Arabia [28] and Jordan [29]. However, 0.4%, 3.1%, 1.4% and 0.9% prevalence of *Shigella* species were reported from Hawassa, Ethiopia [26], Gondar, Ethiopia [27], Haramaya, Eastern Ethiopia [25] and Iran [34] respectively. The prevalence of *Shigella* among food handlers in a community may vary according to geographical region. These differences might be due to the higher standards of education, differences in geographical variation and socio-demographic characteristics of the study population.

In the present study food handlers’ hand washing practices after toilet with soap and water was 68.6% which is in line with the findings from Gondar (63.3%) [35] and Nigeria (71.9%) [36]. However, it is low compared to a report from Jimma (77%) [37] and Addis Ababa (80.8%) [33]. and it is high compared to report from Arba Minch (56.4%) [15]. Compared to finding from Jimma (57%) [37], high proportion of food handlers (75.4%) in this study had a habit of washing with water and soap before touching food.

Based on the present study, 62.3% of food handlers keep their fingernail trimmed which is lower compared to report from Jimma, Ethiopia (80.4%) [37] but a comparable finding was reported from Nigeria (69.1%) [23]. Unlike the current study, (8.9%), high proportion of study participants from Bahr Dar (21.8%) [38] and Jimma 969.6%) [37] received training on ‘food handling and preparation’. The proportion food handlers who had worn appropriate hair cover found in this study (38.6%) is similar with report from Jimma (40.4%) [37]. Moreover, proportion of food handlers who had worn protective coat in this study (82.2%) is comparable with finding from North West Ethiopia (88.7%) [39]. In this study, 37.3% of food handlers had medical checkup, this finding is lower than report from Bahir Dar, Ethiopia (83.5%) and Jimma, Ethiopia (57%) [19, 37].

In this study, hand washing habit and food handling practices were found unsatisfactory. Therefore, it is preferable to combine proper hand washing with the other food hygienic practices in order to prevent food contamination. In general only food handlers hand washing habit after toilet had a significant association with the prevalence of *Salmonella* species (P = 0.03) which is similar with the study in Arba Minch, South Ethiopia [15]; however, a study in Addis Ababa, Ethiopia found no significant association between the prevalence of *Salmonella* and hand washing habit after toilet [33].

In this study 80% of the isolated *Salmonella* species were resistant to tetracycline which is high compared to study from Gondar (46.2%) [39]. The resistance profile to cotrimoxazole and chloramphenicol is comparable with the study done in Debre Markos, Ethiopia [40]. Sixty percent and 20% of isolated *Salmonella* species were resistant to ampicillin and nalidixic acid respectively which is similar with the report from Gondar, Ethiopia [35] but higher resistance to ampicillin was reported from Addis Ababa and Debre Markos [33, 40]. These differences might be due to the low number of isolates in our study, geographical differences and study population.

All the isolated *Salmonella* species were susceptible to ciprofloxacin, and ceftriaxone which are consistent with the study conducted in Nigeria, Jimma, Arba Minch, Debre Markos and Addis Ababa [15, 22, 35, 40–42].

## Limitation

The present study had limited to serotype the isolated *Salmonella* species due to the scarcity of financial and reagents constraints. Using H2S negative as one criterion may have caused false negative as some *Salmonella* are H2S producer.

## Data Availability

The datasets used and analyzed during the current study available from the corresponding author on reasonable request.

## Abbreviations

CDC: Center for Disease Control and Prevention
CLSI: Clinical Laboratory Standard Institute
CSA: Central Statistical Agency
FBDs: food-borne diseases
WHO: World Health Organization
MAC: MacConkey
MHA: Muller Hinton Agar
SNNPR: Southern Nations Nationalities and Peoples Region
TSI: Triple Sugar Iron
XLD: xylose-lysine-deoxycholate
